# Plant Photosynthesis-Irradiance Curve Responses to Pollution Show Non-Competitive Inhibited Michaelis Kinetics

**DOI:** 10.1371/journal.pone.0142712

**Published:** 2015-11-12

**Authors:** Maozi Lin, Zhiwei Wang, Lingchao He, Kang Xu, Dongliang Cheng, Genxuan Wang

**Affiliations:** 1 Fuqing Branch of Fujian Normal University, Fuqing, Fujian Province 350300, Republic of China; 2 College of Life Sciences, Zhejiang University, Hangzhou, Zhejiang Province 310027, Republic of China; 3 Key Laboratory of Humid Subtropical Eco-geographical Process, Fujian Normal University, Ministry of Education, Fuzhou, Fujian Province 350007, Republic of China; Texas A&M University at Galveston, UNITED STATES

## Abstract

Photosynthesis-irradiance (PI) curves are extensively used in field and laboratory research to evaluate the photon-use efficiency of plants. However, most existing models for PI curves focus on the relationship between the photosynthetic rate (Pn) and photosynthetically active radiation (PAR), and do not take account of the influence of environmental factors on the curve. In the present study, we used a new non-competitive inhibited Michaelis-Menten model (NIMM) to predict the co-variation of Pn, PAR, and the relative pollution index (*I*). We then evaluated the model with published data and our own experimental data. The results indicate that the Pn of plants decreased with increasing *I* in the environment and, as predicted, were all fitted well by the NIMM model. Therefore, our model provides a robust basis to evaluate and understand the influence of environmental pollution on plant photosynthesis.

## Introduction

Photosynthesis-irradiance (PI) curves, which show the efficiency and capacity of plant photosynthesis with respect to light intensity, have widely been used in both field and laboratory research to evaluate the influences of abiotic and biotic factors (e.g., nutrient limitation, photo-acclimation) on plant performance, e.g., phytoplankton [1–9], *Alnusrubra* [10], winter wheat [11, 12], *Oriza sativa* [13, 14], *Atriplex hastate* [15], *Alocasia macrorrhiza* [15], *Tidestromia oblongifolia* [15], *Trillium grandiflorum* [16], alga [17], and carrots [18]. Accurate assessment of such relationships is of fundamental importance for understanding the photochemical yield of the process and for studying the responses of plants to environmental changes, such as pollution, temperature, water, and light stresses.

Many models have been used to assess the relationship between the photosynthetic rate (Pn) and photosynthetically active radiation (PAR), including the exponential function (EF, [8, 10]), hyperbolic tangent function (HTF, [1]), nonrectangular hyperbola model (NHM, [11, 13]), rectangular hyperbolic model (RHM, [18]), binomial regression function (BRF, [9, 13]), and the modified model based on the rectangular hyperbolic model (MM, [14]). All of these models, except for the three functions (EF, HTF, and BRF), are derived from the Michaelis-Menten equation [11, 14, 17–19, 20]. Biochemically, photosynthesis is essentially a process of reversible enzymatic reaction kinetics, because the primary process in photosynthesis is an oxidation-reduction reaction [17] and photosynthetic efficiency relies on photon use efficiency by antenna pigments and the catalytic reaction efficiency of CO_2_ by ribulose diphosphate carboxylase. Thus, photons play the role of a resource in photosynthesis, and the relationship between individual gross photosynthesis and PAR can be described by the Michaelis-Menten model [21]. Therefore, the Michaelis-Menten model is optimal to assess the relationship between Pn and PAR. Namely, the RHM, NHM, and MM are all suitable for mathematical fittingof the relationship between Pn and PAR.

However, the PI curve varies significantly with abiotic factors [7], especially environmental pollution [22–25]. Soil pollution, which results from elevated concentrations of pollutants in soil or water, has become a widespread environmental problem because of increased industrialization [26], the land application of sewage sludge [27], and the use of feed additives and/or premixes containing heavy metals in animal husbandry [28]. Thus, it is necessary to build a further model that takes into account the effect of pollution on the relationship between Pn and PAR.

The objectives of the present study were to: 1) build a model for predicting the relationship of Pn, PAR, and *I* (the relative pollution index) in a contaminated environment; and 2) determine why and whether the non-competitive inhibited Michaelis-Menten model (NIMM) is suitable for predicting the PI curve of plant responses to pollution. However, because there are three kinds of pollutant-induced inhibited enzymatic reactions, including competitive, non-competitive, and un-competitive, it is also important to determine which is the most suitable to show the inhibiting effect of pollutants on the PI curve.

## Materials and Methods

### 2.1 The non-competitive inhibited Michaelis-Menten model

Michaelis and Menten [29] proposed the Michaelis-Menten equation (Eq 1) to describe the relationship between v and [S] in enzymatic reactions,

$$\text{v}=\frac{{\text{V}}_{\text{m}}\cdot \left[\text{S}\right]}{{\text{K}}_{\text{m}}+\left[\text{S}\right]}$$ (1)

where v is the velocity of the enzymatic reaction, V_m_ is the maximum velocity of the enzymatic reaction, [S] is the content of the substrate in the enzymatic reaction, and K_m_ is the Michaelis constant. Further, in an inhibitor-induced enzymatic reaction, three general types of inhibition kinetics equations (i.e., competitive, Eq 2; non-competitive, Eq 3; and uncompetitive, Eq 4) can be derived from the Michaelis-Menten equation [29, 30],

$$\text{v}=\frac{{\text{V}}_{\text{m}}\cdot \left[\text{S}\right]}{{\text{K}}_{\text{m}}\cdot \left(1+\frac{\left[I\right]}{{\text{K}}_{\text{i}}}\right)+\left[\text{S}\right]}$$ (2)

$$\text{v}=\frac{{\text{V}}_{\text{m}}\cdot \left[\text{S}\right]}{\left({\text{K}}_{\text{m}}+\left[\text{S}\right]\right)\cdot \left(1+\frac{\left[I\right]}{{\text{K}}_{\text{i}}}\right)}$$ (3)

$$\text{v}=\frac{{\text{V}}_{\text{m}}\cdot \left[\text{S}\right]}{{\text{K}}_{\text{m}}+\left[\text{S}\right]\cdot \left(1+\frac{\left[I\right]}{{\text{K}}_{\text{i}}}\right)}$$ (4)

in these equations, v, V_m_, [S], and K_m_ are the same as mentioned above; [*I*] is the content of the inhibitor; and K_i_ is the inhibition constant. As mentioned above, photosynthesis is a process of enzymatic reactions, and photons play the role of a resource [21], the PAR in photosynthesis is similar to the [S] in an enzymatic reaction.

The RHM (Eq 5) was derived from the Michaelis-Menten equation [11, 14, 18, 19],

$$\text{Pn}=\frac{\alpha \cdot {\text{P}}_{\text{m}}\cdot \text{PAR}}{\alpha \cdot \text{PAR}+{\text{P}}_{\text{m}}}-\text{Rd}$$ (5)

where α is the photochemical efficiency of photosynthesis at low light, P_m_ is the maximum photosynthetic rate, PAR is the photosynthetically active radiation, and Rd is the dark respiration rate.

Ye [14] presented a new model (Eq 6) modified from the RHM (Eq 5) for predicting the relationship between Pn and PAR,

$$\text{Pn}=\frac{\alpha \cdot \left(1-\beta \cdot \text{PAR}\right)\cdot \text{PAR}}{1+\gamma \cdot \text{PAR}}-\text{Rd}$$ (6)

Where α is the photochemical efficiency of photosynthesis at low light, i.e., the initial slope of the PI curve; β is a correction factor for the decreasing trend of Pn when PAR exceed light saturation point due to photoinhibition, and the β is similar to the convexity [9, 11] or the sharpness of the knee [20] of the PI curve, γ is a conversion factor for the α (i.e., the initial slope of the PI curve) and the P_m_ (i.e., the maximum photosynthetic rate), and the γ is proportional to the radio of α and P_m_ (i.e., $\gamma \propto \frac{\alpha }{{\text{P}}_{\text{m}}}$); α, β, and γare coefficients that are independent of irradiance [14]; PAR is the photosynthetically active radiation, and Rd is the dark respiration rate. Here, we assumed that, 1) the Pn of plants decreased with increasing concentrations of a pollutant; and 2) the effect of the pollutant on the PI curve is non-competitive inhibited, and we presented our new non-competitive inhibited Michaelis-Menten model (NIMM) as:

$$\text{Pn}=\frac{\alpha \cdot \left(1-\beta \cdot \text{PAR}\right)\cdot \text{PAR}}{\left(1+\gamma \cdot \text{PAR}\right)\cdot \left(1+\frac{I}{{\text{K}}_{\text{i}}}\right)}-\text{Rd}$$ (7)

Where α, β and γare the same as mentioned above; Pn denotes the net photosynthetic rate; K_i_ denotes an inhibition constant; *I* is the relative pollution index and

$$I=\frac{{\text{C}}_{\text{i}}}{{\text{C}}_{\text{imax}}}$$ (8)

Where C_i_ is the actual concentration of pollutant i in water or soil; and C_imax_ is the maximum concentration of pollutant i in water or soil.

### 2.2 Experimental design

Establishing a single pollutant model is the first step in the research of effects of pollution on plants. Here, we chose one pollutant to a plant research model. We tested effects of a variety of common pollutants to corresponding representative plants as shown in Table 1. Phenolic pollution is often the chemical hazards and accidents that take place in the chemical industry. And the soil heavy metal pollution result from rapid industrialization and urbanization during industrial and agricultural development and population growth. So, we tested the pollutants including phenol and some common metal pollutants, e.g., Cu^2+^, Pb^2+^, Cd^2+^, and Al^3+^. The Bordeaux mixture (a mixture of coppersulfate and lime) or animal manure use in agriculture results in the potential risk of soil copper pollution. The lead and cadmium pollution also result from automobile exhaust. The soil acidity increasing leads to aluminum pollution. The plants we considered including monocotyledonous or dicotyledonous plant, C_3_ or C_4_ plant, herbaceous or woody plant, or crop. We collected and analyzed the data of effects of phenol and Cu^2+^ on plants from pot-culture experiments. For additional information, we also extracted and analyzed the data about the effects of other pollutants such as Pb^2+^, Cd^2+^ and Al^3+^ on plants from published literatures [22–25].

**Table 1:** Data matrix for model establishing.

Species	Species types	pollutant	Data source
*Trifolium pratense*	monocotyledonous, herbaceous, C_3_ plant	phenol	Measured in this study
*Wedelia trilobata*	dicotyledonous, herbaceous, C_3_ plant	Cu^2+^	Measured in this study
*Zea mays*	monocotyledonous, crop, C_4_ plant	Pb^2+^	Data collected from literature [22]
*Citrus sinensis* Osbeck	dicotyledonous, woody, C_3_ plant	Cu^2+^	Data collected from literature [23]
*Zea mays*	monocotyledonous, crop, C_4_ plant	Cd^2+^	Data collected from literature [24]
*Plantago asiatica*	dicotyledonous, herbaceous, C_3_ plant	Al^3+^	Data collected from literature [25]

### 2.3 Pot-culture experiment and PI curves measurement

The pot-culture experiments were carried out in a greenhouse at the Fuqing Branch of Fujian Normal University from June to September in 2013. *T*. *pratense* L. and *W*. *trilobata*, two types of ornamental groundcover that often appear on roadsides and plantations, were planted in flowerpots filled with ≈ 1.8 kg soil. Each treatment had 15 replicates. The properties of the soil were pH: 6.4, total nitrogen: 24.2 mg kg^-1^, total phosphorus: 1.15 g kg^-1^, available phosphorus: 9.03 mg kg^-1^, total potassium: 68 mg kg^-1^, and clay particles: 21.7%.

*T*. *pratense* seeds were germinated for 48 h in the dark (on wet filter paper at 25°C) and sown into a flowerpot (diameter: 200mm, height: 200mm) filled with phenol treated soil. Before being filled into pot, air-dried soil was treated with 0 (as control), 100, 200, or 300 mg kg^-1^ of phenol. *W*. *trilobata* were collected from the roadsides and cut, and the apex meristem with two leaves (≈ 100-mm length, two internodes) were planted in a flowerpot (diameter: 200 mm, height: 200 mm). Three apex meristems were planted in every flowerpotwith CuSO_4_·5H_2_O added soil. Air-dried soil was added with 0 (as control), 500, 1000, or 2000 mg kg^-1^ of CuSO_4_·5H_2_O, and then was filled into the flower port.

We selected a sunny day (three months after planting) to measure the PI curves using a CIRAS-2 Portable Photosynthesis System (PP Systems, USA) with an LED radiation source.

### 2.4 Data collection and detailed data descriptions

PI data for plants under different concentrations of pollutants from four studies were gathered from published literatures (Table 1) to further evaluate our NIMM. All data were collected from pot-culture experiments.

The pot-culture experiments of *Z*. *mays* seedling [22] were conducted in silica culture. And the seedlings consisting of one bud and two leaves were treated with three Hoagland solution (including equal amount of Pb^2+^ and EDTA at different concentrations: 0, 0.25 or 0.5 mmol·L^-1^). After 15 days of treatment, the PI curves were measured with a Ciras-2 portable photosynthesis system (PP systems, UK). For more detailed information, please see S1 Table.

The one-year old *C*.*sinensis* Osbeck [23] was grafted onto *Citrus aurantium* L. before Cu stress treatment. The pot-culture experiments of *C*.*sinensis* Osbeck were conducted in a 10-L pot filled with 8 L of Alva nutrient solution (pH 6.5). The Alva nutrient solution was aerated 3 times with each time for 2 h in every day, and it was renewed every 10 days; And the *C*.*sinensis* Osbeck were treated with five Alva nutrient solution (containing Cu^2+^ concentration at0, 0.1, 5, 20 or 40 μmol·L^-1^). After 60 days of treatment, the PI curves were measured with a CID-301 PS (CID Bio-Science, Inc., USA). For more detailed information, please see S2 Table.

The other pot-culture experiments of *Z*. *mays* [24] were conducted in paddy soil. The properties of the paddy soil were pH: 6.42, organic matter: 1.63%, total Cd: 0.32 mg·kg^-1^, total nitrogen: 0.09%, available phosphorus: 0.05%, available potassium: 0.04%. The paddy soil was air-dried and sieved through a 2-mm sieve, mixed with different amount of CdCl_2_·2.5H_2_O, and then the post-treated paddy soil was added into each pot up to three kg with one gram of compound fertilizer (including N 15%, P 15%, K 15%). Finally, the germinated *Z*. *mays* were planted; So far, the germinated *Z*. *mays* were treated with six paddy soil (including Cd concentration at: 0.32, 1, 5, 15, 50 or 100 mg·kg^-1^). After 20 days of treatment, the PI curves were measured with Li-6400 (Li-Cor Inc., USA). For more detailed information, please see S3 Table.

The *P*. *asiatica* [25] seed was sterilized with 0.1% HgCl_2_ for 10 min, following by washing and soaking in distilled water for 8 h, and then the seed was sowed in sterilized silica culture. The two-leaves old plantswere transplanted into a 20 cm × 23cm flowerpot with three kg medium (peat soil: sand = 3:1). On the six-leaves old plant, the Al stress was performed. 10 mL of AlCl_3_ solution (pH 4.0) with different concentration at 0, 100, 500, 800 or 2000 mg·L^-1^ were respectively poured into the flowerpot to simulate different leaching of Al^3+^ in soil every day. After 20 days of treatment, the PI curves were respectively measured with a Ciras-2 portable photosynthesis system (PP systems, UK). For more detailed information, please see S4 Table.

### 2.5 Mathematical fitting and model testing

To obtain the equation parameters (*i*.*e*., α, β, γ, K_i_, and Rd), mathematical fitting of NIMM was performed using 1stOpt software (7D-Soft High Technology Inc. Beijing, China) with the Levenberg-Marquardt method. In addition, mathematical fitting of the relationship of Pn and *I* and that of Pn and PAR were performed to obtain the equation parameters using the same software and method as in the previous case. The relationship between the Pn and PAR of *T*. *pratense* response to different concentrations of phenol in our pot-culture experiment was calculated according to the mathematical fitting results to test the NIMM. The relationship between the Pn and PAR of *W*. *trilobata* response to different concentrations of Cu^2+^ was calculated using the same method.

## Results

### 3.1 Experimental results

The Pn in our pot-culture experiments was measured with a Ciras-2 under conditions of natural ambient CO_2_ at different PAR. Our results were similar to the references [22–25]. Clearly, the PI curves of the plants were saturation curves. The results also showed that, either in *W*. *trilobata* or in *T*. *pratense*, the Pn increased with PAR increasing below the PAR_sat_ (i.e., light saturation point, ≈ 1000 μmol photon m^-2^ s^-1^ in *T*. *pratense*, ≈ 1400 μmol photon m^-2^ s^-1^ in *W*. *trilobata*), while decreased as PAR increasing above PAR_sat_. The results also showed that the pollutant obviously negatively affected the PI curves. For more detailed information, please see S5 and S6 Tables.

### 3.2 Effect of a pollutant on the normalized Pn of plants

**Fig 1:**
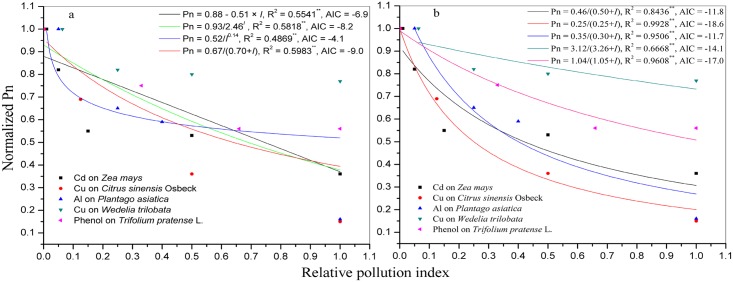
Effect of a pollutant on the normalized Pn under 1000 μmolphotonm^-2^ s^-1^ PAR. a, the normalized Pn of all five species regressed with respect to *I* using linear, power, exponential, and hyperbolic functions. b, the normalized Pn of each species regressed with respect to *I* using the hyperbolic function. AIC is Akaike's information criterion. ** means significant at *P* ≤ 0.01.

The normalized Pn of plants decreased with increasing concentrations of the pollutant under 1000 μmol photon m^-2^ s^-1^ PAR (Fig 1). Akaike's information criterion (AIC) was proposed by Akaike [31, 32] and defined as Eq 9,

$$\text{AIC}=\text{N}\cdot \text{ln}{\text{R}}_{\text{e}}+2\cdot \text{p}$$ (9)

Where N is the number of experimental data points, p is the number of parameters in an estimated model, and R_e_ is the residual sum of squares. In addition, the model with the lowest AIC is regarded as the best representation of a curve [32]. The Pn values for all five species were normalized to the pollutant-free control value of Pn, and the normalized Pn were regressed with respect to *I* using linear (Eq 10), power (Eq 11), exponential (Eq 12), and hyperbolic (Eq 13) functions,

$${\text{Pn}}^{\prime }=\text{a}+\text{b}\times I$$ (10)

$${\text{Pn}}^{\prime }=\frac{\text{a}}{I^{\text{b}}}$$ (11)

$${\text{Pn}}^{\prime }=\frac{\text{a}}{{\text{b}}^{I}}$$ (12)

$${\text{Pn}}^{\prime }=\frac{\text{a}}{\text{b}+I}$$ (13)

in these equations (Eqs 10 ~ 13), Pn′ is the normalized net photosynthetic rate, a and b are coefficients, *I* is the relative pollution index.

And the results showed that all functions (Eqs 10, 11, 12 and 13) were significant (*P*< 0.01), and the hyperbolic function (Eq 13) was the optimal function based on having the greatest goodness-of-fit (R^2^) of 0.5983 and the lowest AIC of -9.0 (Fig 1a). The normalized Pn of each species was regressed with respect to *I* using a hyperbolic (Eq 13) function, and the results were all significant (*P*< 0.01) (Fig 1b).

### 3.3 Mathematical fitting of PI curves using different models

**Fig 2:**
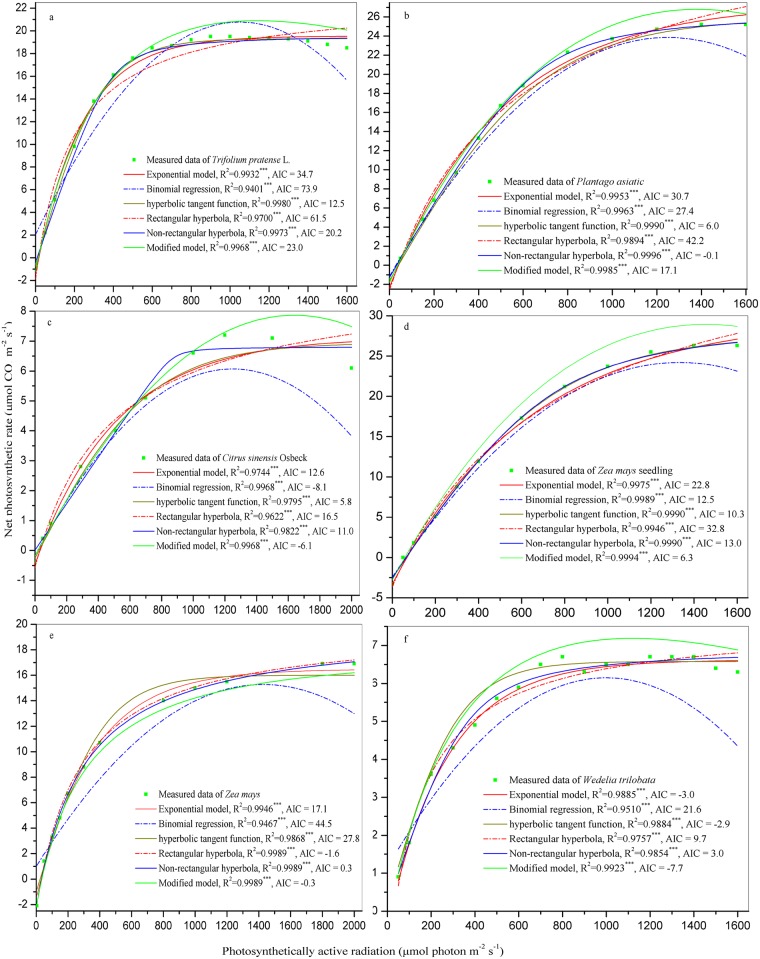
Mathematical fitting of the PI curve using different models. AIC is Akaike's information criterion.

The Pn of *T*. *pratense*, *Z*. *mays* seedling, *C*. *sinensis* Osbeck, *Z*. *mays*, *P*. *asiatica*, *W*. *trilobata* were respectively regressed with respect to PAR usingan EF [8, 10], HTF [1], NHM [11, 13], RHM [17, 18], BRF [9,13], and MM [14]. The R^2^ was significant for all models (*P*< 0.001). In *T*. *pratense* or *P*. *asiatica*, the three largest R^2^ values (associated with the lowest AIC) of models were for HTF, NHM, and MM (Fig 2a and 2b). In *C*. *sinensis* Osbeck or *Z*. *mays* seedling, the three largest R^2^ values (associated with the lowest AIC) of models were for HTF, BRF, and MM (Fig 2c and 2d). In *Z*. *mays*, the three largest R^2^ values (associated with the lowest AIC) of models were for RHM, NHM, and MM (Fig 2e). In *W*. *trilobata*, the three largest R^2^ values (associated with the lowest AIC) of models were for EF, HTF, and MM (Fig 2f). The MM and BRF were both better than other models at describing the photoinhibition phenomenon at high PAR (Fig 2).

### 3.4 Evaluation of NIMM

The Pn of each species was regressed on PAR and *I* using NIMM, and the results are shown in Table 2. The R^2^ values were greater than 0.95 except for *Z*. *mays*. For Cu pollution, the K_i_ of *W*. *trilobata* was greater than that of *Citrus sinensis* Osbeck. The K_i_ of Cu to *W*. *trilobata* was greater than that of the phenol to *T*. *pratense*. For *Z*. *mays*, the K_i_ of Cd was greater than that of Pb.

**Table 2:** Mathematical fitting results of the NIMM for plant responses to pollution.

		Model parameters					
Species (Pollutant)	Data source	K_i_	α	β	γ	Rd	R^2^
*Trifolium pratense* (Phenol)	Measured in this study	1.17	0.086	0.0002	0.0022	1.03	0.9886
*Wedelia trilobata* (Cu)	Measured in this study	4.48	0.044	0.0001	0.0042	1.00	0.9629
*Zea mays* (Pb)	Reference [22]	0.395	0.044	0.0003	0.0002	1.78	0.9841
*Citrus sinensis* Osbeck (Cu)	Reference [23]	0.321	0.013	0.0003	0.0002	0.42	0.9862
*Zea mays* (Cd)	Reference [24]	0.923	0.061	0.0001	0.0015	1.65	0.8984
*Plantago asiatica* (Al)	Reference [25]	0.501	0.058	0.0003	0.0005	1.59	0.9576

K_i_ denotes the inhibition constant; α denotes the photochemical efficiency of photosynthesis at low light, i.e., the initial slope of the PI curve; β and γ are the coefficients that are independent of irradiance; Rd denotes the dark respiration rate.

**Fig 3:**
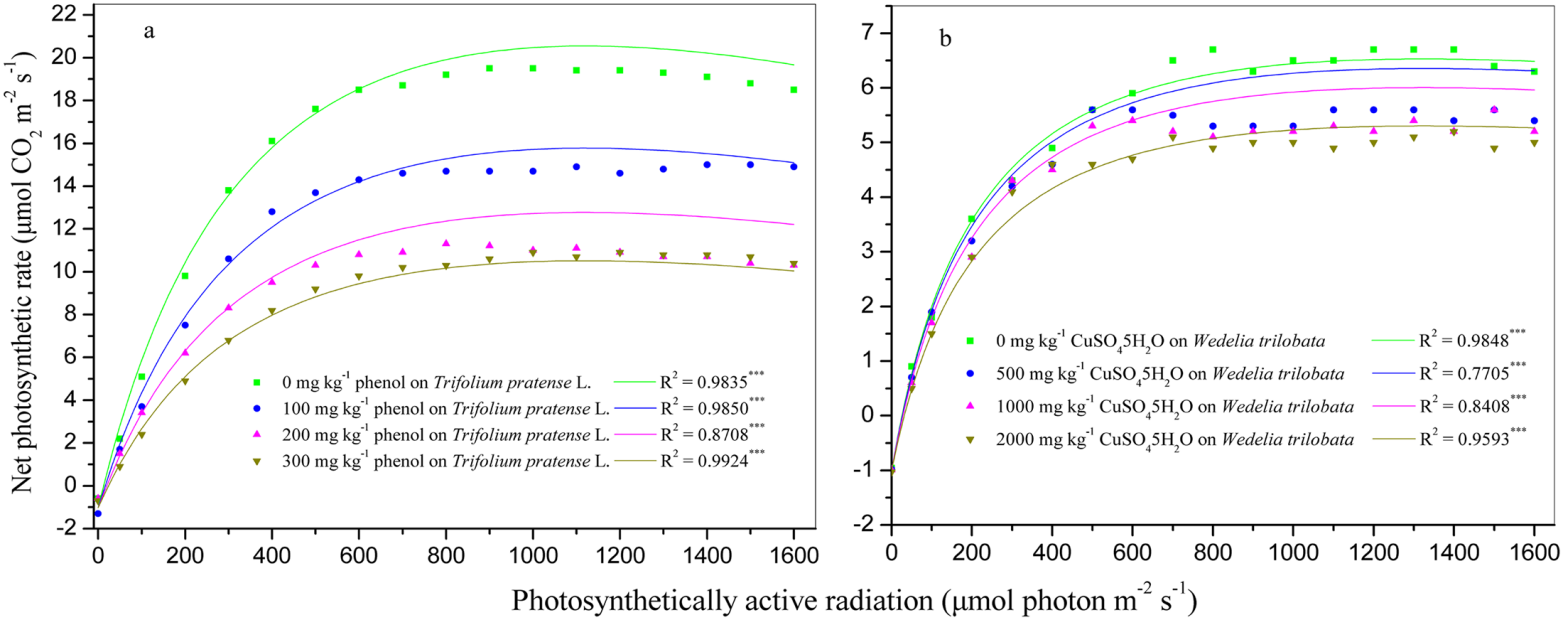
The test results for the NIMM. a, in *T*. *pratense*; b, *in W*. *trilobata*; *** means significant at *P* ≤ 0.001.

The NIMM was tested using our pot-culture experimental data. Either in *T*. *pratense*, or in *W*. *trilobata*, the R^2^ values were all significant (*P*< 0.001) under different pollution levels (Fig 3, Table 3). Either in *T*. *pratense*, or in *W*. *trilobata*, the light saturation point (PAR_sat_) and the light compensation point (PAR_com_) both increased with worsening pollution, while the maximum photosynthetic rate (P_m_), quantum efficiency at PAR_com_ (φ_c_), and intrinsic quantum efficiency (φ_0_) all decreased (Table 3). The φ_c_ represents the light energy use efficiency at PAR_com_, the φ_0_ represents the intrinsic light energy use efficiency at darkness, i.e., the optimal light use potential of plant. The results suggested that the pollutant inhibited the light use potential of plant. In order to analyze the credibility of the assessment results, we performed paired samplest test analysis, and the results showed that in *T*. *pratense*, the calculated P_m_ was no significant difference to the measured P_m_ (*t* = -1.975, *df* = 3, *P*_2-tailed_ = 0.143), in *W*. *trilobata*, the calculated P_m_ was also no significant difference to the measured P_m_ (*t* = -1.777, *df* = 3, *P*_2-tailed_ = 0.174).

**Table 3:** Model testing results of the NIMM.

Species	Pollutant in soil (mg kg^-1^)	Calculated equation	Measured P_m_ (μmolCO_2_m^-2^ s^-1^)	Calculated P_m_ (μmolCO_2_m^-2^ s^-1^)	PAR_com_(μmolphoton m^-2^ s^-1^)	PAR_sat_(μmolphoton m^-2^ s^-1^)	φ_c_	φ_0_	R^2^
*T*. *pratense*									
	Phenol (0)	$\text{Pn}=\frac{0.086\cdot \left(1-0.0002\cdot \text{PAR}\right)\cdot \text{PAR}}{1+0.0022\cdot \text{PAR}}-1.03$	19.5	20.5	12.0	1140.7	0.083	0.089	0.9835***
	Phenol (100)	$\text{Pn}=\frac{0.067\cdot \left(1-0.0002\cdot \text{PAR}\right)\cdot \text{PAR}}{1+0.0022\cdot \text{PAR}}-1.03$	15.0	15.8	15.4	1146.2	0.065	0.070	0.9850***
	Phenol (200)	$\text{Pn}=\frac{0.055\cdot \left(1-0.0002\cdot \text{PAR}\right)\cdot \text{PAR}}{1+0.0022\cdot \text{PAR}}-1.03$	11.3	12.8	18.7	1152.2	0.053	0.057	0.8708***
	Phenol (300)	$\text{Pn}=\frac{0.046\cdot \left(1-0.0002\cdot \text{PAR}\right)\cdot \text{PAR}}{1+0.0022\cdot \text{PAR}}-1.03$	10.9	10.6	22.2	1158.1	0.044	0.049	0.9924***
*W*. *trilobata*									
	CuSO_4_·5H_2_O (0)	$\text{Pn}=\frac{0.044\cdot \left(1-0.0001\cdot \text{PAR}\right)\cdot \text{PAR}}{1+0.0042\cdot \text{PAR}}-1.00$	6.7	6.7	23.0	1397.0	0.040	0.048	0.9848***
	CuSO4·5H2O (500)	$\text{Pn}=\frac{0.042\cdot \left(1-0.0001\cdot \text{PAR}\right)\cdot \text{PAR}}{1+0.0042\cdot \text{PAR}}-1.00$	5.6	6.3	24.0	1400.0	0.038	0.046	0.7705***
	CuSO_4_·5H_2_O (1000)	$\text{Pn}=\frac{0.040\cdot \left(1-0.0001\cdot \text{PAR}\right)\cdot \text{PAR}}{1+0.0042\cdot \text{PAR}}-1.00$	5.6	5.9	25.3	1404.0	0.036	0.044	0.8408***
	CuSO_4_·5H_2_O (2000)	$\text{Pn}=\frac{0.036\cdot \left(1-0.0001\cdot \text{PAR}\right)\cdot \text{PAR}}{1+0.0042\cdot \text{PAR}}-1.00$	5.2	5.3	27.8	1411.8	0.032	0.040	0.9593***

PAR_sat_ is light saturation point; PAR_com_ is light compensation point; P_m_ is maximum photosynthetic rate; φ_c_ is the quantum efficiency at PAR_com_; φ_0_ is intrinsic quantum efficiency; ${\text{PAR}}_{\text{com}}=\frac{\text{Rd}}{\alpha }$, φ_0_ = α∙[1+(β+γ)∙PAR_com_], $\varphi_{\text{c}}=\alpha \cdot \frac{1+\left(\beta +\gamma \right)\cdot {\text{PAR}}_{\text{com}}-\beta \cdot \gamma \cdot {\text{PAR}}_{\text{com}}^{2}}{{\left(1+\gamma \cdot {\text{PAR}}_{\text{com}}\right)}^{2}}$, ${\text{PAR}}_{\text{sat}}=\frac{\sqrt{\frac{\left(\beta +\gamma \right)\cdot \left(1+\gamma \cdot {\text{PAR}}_{\text{com}}\right)}{\beta }}-1}{\gamma }$, ${\text{P}}_{\text{m}}=\frac{\alpha \cdot \left(1-\beta \cdot {\text{PAR}}_{\text{sat}}\right)\cdot {\text{PAR}}_{\text{sat}}}{1+\gamma \cdot {\text{PAR}}_{\text{sat}}}-\text{Rd}$;

*** means significant at *P* ≤ 0.001.

## Discussion

All of the above mentioned existing models (i.e., EF; HTF; NHM; RHM; BRF; and MM) provide useful protocols for PI curve assessment. Jassby and Platt reported that, from zero light up to the onset of photoinhibition, the PI curve for natural populations of coastal phytoplankton is best described by HTF, and they recommended its use as an operational model for the elucidation of physiological parameters in photosynthesis-light experiments and for the theoretical investigation [1]. The shape of PI curve described by EF suggests that a linear relation holds only for low light intensities, then the photosynthetic rate tends towards a maximum valuewhen the light intensity is increasing [8, 10]. The NHM was found to be objective to calculate the photosynthetic parameters of the PI curve [9, 11, 13, 20], the PI curve could also be described by BRF [13, 33], but the BRF could not be used to calculate thequantum efficiencyand explain that the predicted Pn declines quickly when PAR excesses the light saturation point [13]. In addition, the BRF has the shortcoming of sometimes inferring a positive dark respiration rate, which has no biological significance. The RHM can be obtained from the NHM by putting θ = 0, it is a special case of the NHM [20]. And the RHM is preferred to the NHM by some workers on the grounds of simplicity [18, 20], though it is rather tedious to take the limit as θ → 0 in the NHM equation [20]. Our experimental results showed that the shapes of PI curves were similar to that of the literatures. Our experimental results also showed that the PI curves have photoinhibition phenomenon at high irradiance, i.e., the Pn decreased when the PAR exceeded light saturation point. These results were fully consistent with that of the literature [8, 11, 13, 14, 23, 34]. Although the HTM, EF, NHM and RHM have been extensively applied [11, 14, 17, 18, 20, 34–38], they do not consider the photoinhibition of plants. The MM, which is based on the RHM, is useful to study photoinhibition and photosynthetic behavior at high irradiance and, especially, is the best model to describe the PI curve because its fitted values wereclose to the measured data [14]. Therefore, the MM (Eq 6) was the optimal modelfor predicting the relationship of Pn and PAR. Moreover, based on the lowest AIC values [31, 32], the HTF, NHM, and MM are more suitable for characterizing the PI curve (Fig 2).

Temperature, intensity of irradiation, and concentration of carbon dioxide in the surrounding medium are the three important controlling factors could influence the rate of photosynthesis in plant, and of the three controlling factors, the most important is the temperature [11, 17]. However, the concentration of carbon dioxide in the atmosphere remains relatively constant, and it is unlikely to be a major factor effecting variations in the rate of photosynthesis, simultaneously, the temperature could not influence the shape of the PI curve of plant, therefore, temperature and concentration of carbon dioxide need not appear explicitly in a PI curve model [11]. On the other hand, the shapes of PI curves in our pot-culture experiments (Fig 3) were fully consistent with that of the literatures [8, 11, 13, 14, 22–25, 34], and showed that α and P_m_ both decreased along with the increasing concentrations of pollutant, but the convexity [11, 13, 37], or the sharpness of the knee [20] of the PI curve described by the NHM increased along with increasing pollutant concentrations. It indicated that the pollutants negatively affected on the photosynthesis of plants, and the impact degree increased with rising pollutant concentrations. This conclusion was similar to that of the literature [17]. The literature [17] reported that a poison may materially to reduce the rate of photosynthesis, because the poison may either decrease the velocity of the Blackman reaction, or decrease the velocity of the primary photosynthetic reaction by being preferentially adsorbed by the chlorophyll a and thus preventing the latter from adsorbing or combining with hydrated carbon dioxide. So, pollutant was significant and necessary appear explicitly in a PI curve model. And even though some metals, such as zinc and copper, are essential trace elements for plants as the natural active sites of an enzyme, plant growth and development only need low concentrations of these metals of around 10 μg g^-1^ dry plant tissue [39, 40]. Some studies [41, 42] have also shown that pollutants (heavy metals) significantly affect the Pn of plants. Hence, in the present study, an attempt was made to build a new model, which was integrated *I* (i.e., pollution index) into the MM, for predicting the relationship of Pn, PAR and *I*.

Then, how to integrate the *I* into the MM? The relationship of normalized Pn and *I* were respectively regressed using linear (Eq 10), power (Eq 11), exponential (Eq 12), and hyperbolic (Eq 13) functions. And, the effect of pollutants on the Pn of plants (Fig 1) indicated that the hyperbolic function (Eq 13) was optimal for predicting the relationship of Pn and *I*. Thus, we integrated the *I* into the MM as:

$$\text{Pn}=\frac{\alpha \cdot \left(1-\beta \cdot \text{PAR}\right)\cdot \text{PAR}}{1+\gamma \cdot \text{PAR}}\cdot \frac{\text{a}}{\text{b}+I}-\text{Rd}$$ (14)

Eq 14 can be converted into:

$$\text{Pn}=\frac{\alpha \cdot \left(1-\beta \cdot \text{PAR}\right)\cdot \text{PAR}}{(1+\gamma \cdot \text{PAR})\cdot (\frac{\text{b}+I}{\text{a}})}-\text{Rd}$$ (15)

Further, Eq 15 can be converted into:

$$\text{Pn}=\frac{\alpha \cdot \left(1-\beta \cdot \text{PAR}\right)\cdot \text{PAR}}{(1+\gamma \cdot \text{PAR})\cdot \frac{\text{b}}{\text{a}}\cdot (1+\frac{I}{\text{b}})}-\text{Rd}$$ (16)

If b = K_i_ and $\delta =\frac{\text{b}}{\text{a}}$, Eq 16 can be expressed as:

$$\text{Pn}=\frac{\alpha \cdot \left(1-\beta \cdot \text{PAR}\right)\cdot \text{PAR}}{(1+\gamma \cdot \text{PAR})\cdot \delta \cdot (1+\frac{I}{{\text{K}}_{\text{i}}})}-\text{Rd}$$ (17)

Where δ is a non-zero coefficient, Eq 17 is equivalent to the NIMM, i.e., Eq 7.

Further, our mathematical fitting results showed that the NIMM was suitable for predicting the relationship of Pn, PAR, and *I* because of their high R^2^ (Table 2) and their significance at the *P*< 0.001 level (Table 3), that is, the NIMM was suitable for fitting the PI curve of plant responses to pollution (Fig 3, Table 3). The NIMM showed that the Pn is a function of PAR and *I*, thus, the Eq 18 denotes the influence rate of *I* on Pn, and the Eq 19 denotes the influence rate of PAR on Pn,

$$\frac{\partial \text{Pn}}{\partial I}=-\frac{\alpha \cdot \left(1-\beta \cdot \text{PAR}\right)\cdot \text{PAR}}{{\text{K}}_{\text{i}}\cdot \left(1+\gamma \cdot \text{PAR}\right)\cdot {\left(1+\frac{I}{{\text{K}}_{\text{i}}}\right)}^{2}}$$ (18)

$$\frac{\partial \text{Pn}}{\partial \text{PAR}}=\frac{\alpha -2\cdot \alpha \cdot \beta \cdot \text{PAR}-\alpha \cdot \beta \cdot \gamma \cdot {\text{PAR}}^{2}}{{\text{K}}_{\text{i}}\cdot {\left(1+\gamma \cdot \text{PAR}\right)}^{2}\cdot \left(1+\frac{I}{{\text{K}}_{\text{i}}}\right)}$$ (19)

Where $\frac{\partial \text{Pn}}{\partial I}$ and $\frac{\partial \text{Pn}}{\partial \text{PAR}}$ are partial derivative, denotes the influence rate of *I* on Pn, and the influence rate of PAR on Pn respectively; α, β, γ, K_i_, PAR, and *I* are the same as above mentioned.

In all the published models (i.e., HTF [1], EF [8], EF [10], NHM [11, 13], BRF [9, 13], RHM [17, 18]), the researchers focused more on the relationship between the Pn and PAR, however, they didn’t take account of the influence of *I* on the PI curve. In the present study, we have integrated the *I* into the MM [14] as the NIMM topredict the co-variation of Pn, PAR, and the *I*. Here, we also integrated the *I* into the published models (i.e., HTF [1], EF [8], EF [10], NHM [11, 13], BRF [9, 13], RHM [17, 18], respectively) to predict the co-variation of Pn, PAR, and the *I*. Then, we compared the NIMM with the modified models based on our pot-culture experimental data (Table 4). In *T*. *pratense*, the AIC of the NIMM (i.e., 242.5) was lower than that of the models which were modified from the EF [8, 10], RHM [17, 18], and BRF [9, 13] (i.e., 277.0, 249.6, 308.2, and 357.3 respectively), while, the AIC of the NIMM was higher than that of the models modified from the HTF [1] or NHM [11, 13] (i.e., 229.0 or 235.3 respectively). In *W*. *trilobata*, the AIC of the NIMM (i.e., 131.2) was lower than that of the models which were modified from the EF [8], RHM [17, 18], and BRF [9, 13] (i.e., 164.8, 151.5, and 209.2 respectively), while, the AIC of the NIMM was higher than that of the models modified from the EF [10], HTF [1] or NHM [11, 13] (i.e., 126.4, 124.1, and 128.4 respectively). Although the model with the lowest AIC is regarded as the best representation of a curve [32], the models of the EF [10], HTF [1], NHM [11, 13], and RHM [17, 18] cannot fit the data that shows the photoinhibition phenomenon at high irradiance. The NIMM modified from the MM [14], is more reliable at unveiling the photoinhibition phenomenon. Therefore, the NIMM provides a robust tool to evaluate and understand the influence of environmental pollution on plant photosynthesis, and it is relative improved model comparing to the previous models published [1, 8–11, 13, 17, 18, 20].

**Table 4:** The comparation of model application results in *T*. *pratense* or *W*. *trilobata*.

			Parameters				
Species	Published model	The model equation modified from the published model	Ki	Rd	others	R^2^	AIC
*T*. *pratense*							
	EF, [8]	$\text{Pn}=\frac{\text{a}\cdot \text{PAR}\cdot \text{exp}(\frac{-\text{a}\cdot \text{PAR}}{{\text{P}}_{\text{m}}\cdot e})}{1+\frac{\text{I}}{{\text{K}}_{\text{i}}}}-\text{Rd}$	1.06	0.05	a = 0.05, P_m_ = 20.3	0.9811	277.0
	EF, [10]	$\text{Pn}=\frac{{\text{P}}_{\text{m}}\left(1-\text{exp}\left(\frac{-\text{a}\cdot \text{PAR}}{{\text{P}}_{\text{m}}\cdot e}\right)\right)}{1+\frac{\text{I}}{{\text{K}}_{\text{i}}}}-\text{Rd}$	1.19	1.18	a = 0.24, P_m_ = 20.6	0.9870	249.6
	HTF, [1]	$\text{Pn}=\frac{{\text{P}}_{\text{m}}\cdot \text{tanh}(\frac{\text{a}\cdot \text{PAR}}{{\text{P}}_{\text{m}}})}{1+\frac{\text{I}}{{\text{K}}_{\text{i}}}}-\text{Rd}$	1.13	0.70	P_m_ = 19.9, a = 0.06	0.9903	229.0
	NHM, [11, 13]	$\text{Pn}=\frac{\alpha \cdot \text{PAR}+{\text{P}}_{\text{m}}-\sqrt{{\left(\alpha \cdot \text{PAR}+{\text{P}}_{\text{m}}\right)}^{2}-4\cdot \theta \cdot \alpha \cdot \text{PAR}\cdot {\text{P}}_{\text{m}}}}{2\cdot \theta \cdot \left(1+\frac{\text{I}}{{\text{K}}_{\text{i}}}\right)}-\text{Rd}$	1.11	0.46	P_m_ = 20.1, α = 0.05, θ = 0.9463	0.9897	235.3
	RHM, [17, 18]	$\text{Pn}=\frac{\alpha \cdot \text{PAR}\cdot {\text{P}}_{\text{m}}}{\left(\alpha \cdot \text{PAR}+{\text{P}}_{\text{m}}\right)\cdot \left(1+\frac{I}{{\text{K}}_{\text{i}}}\right)}-\text{Rd}$	1.23	1.52	α = 0.13, P_m_ = 24.3	0.9708	308.2
	BRF, [9, 13]	$\text{Pn}=\frac{\text{a}\cdot {\text{PAR}}^{2}+\text{b}\cdot \text{PAR}}{1+\frac{I}{{\text{K}}_{\text{i}}}}-\text{Rd}$	0.92	-1.39	a = -1.73, b = 0.0371	0.9422	357.3
	NIMM, modified based on MM [14]	$\text{Pn}=\frac{\alpha \cdot \left(1-\beta \cdot \text{PAR}\right)\cdot \text{PAR}}{\left(1+\gamma \cdot \text{PAR}\right)\cdot \left(1+\frac{I}{{\text{K}}_{\text{i}}}\right)}-\text{Rd}$	1.17	1.03	α = 0.086, β = 0.0002, γ = 0.0022	0.9886	242.5
*W*. *trilobata*							
	EF, [8]	$\text{Pn}=\frac{\text{a}\cdot \text{PAR}\cdot \text{exp}(\frac{-\text{a}\cdot \text{PAR}}{{\text{P}}_{\text{m}}\cdot e})}{1+\frac{\text{I}}{{\text{K}}_{\text{i}}}}-\text{Rd}$	3.17	-0.46	a = 0.02, P_m_ = 6.1	0.9372	164.8
	EF, [10]	$\text{Pn}=\frac{{\text{P}}_{\text{m}}\left(1-\text{exp}\left(\frac{-\text{a}\cdot \text{PAR}}{{\text{P}}_{\text{m}}\cdot e}\right)\right)}{1+\frac{\text{I}}{{\text{K}}_{\text{i}}}}-\text{Rd}$	4.25	0.73	a = 0.09, P_m_ = 7.0	0.9643	126.4
	HTF, [1]	$\text{Pn}=\frac{{\text{P}}_{\text{m}}\cdot \text{tanh}(\frac{\text{a}\cdot \text{PAR}}{{\text{P}}_{\text{m}}})}{1+\frac{\text{I}}{{\text{K}}_{\text{i}}}}-\text{Rd}$	3.76	0.17	P_m_ = 6.4, a = 0.02	0.9655	124.1
	NHM, [11, 13]	$\text{Pn}=\frac{\alpha \cdot \text{PAR}+{\text{P}}_{\text{m}}-\sqrt{{\left(\alpha \cdot \text{PAR}+{\text{P}}_{\text{m}}\right)}^{2}-4\cdot \theta \cdot \alpha \cdot \text{PAR}\cdot {\text{P}}_{\text{m}}}}{2\cdot \theta \cdot \left(1+\frac{\text{I}}{{\text{K}}_{\text{i}}}\right)}-\text{Rd}$	3.67	0.06	P_m_ = 6.5, α = 0.02, θ = 0.9200	0.9644	128.4
	RHM, [17, 18]	$\text{Pn}=\frac{\alpha \cdot \text{PAR}\cdot {\text{P}}_{\text{m}}}{\left(\alpha \cdot \text{PAR}+{\text{P}}_{\text{m}}\right)\cdot \left(1+\frac{I}{{\text{K}}_{\text{i}}}\right)}-\text{Rd}$	5.71	2.36	α = 0.09, P_m_ = 9.4	0.9484	151.5
	BRF, [9, 13]	$\text{Pn}=\frac{\text{a}\cdot {\text{PAR}}^{2}+\text{b}\cdot \text{PAR}}{1+\frac{I}{{\text{K}}_{\text{i}}}}-\text{Rd}$	2.47	-1.25	a = -4.68, b = 0.01	0.8795	209.2
	NIMM, modified based on MM [14]	$\text{Pn}=\frac{\alpha \cdot \left(1-\beta \cdot \text{PAR}\right)\cdot \text{PAR}}{\left(1+\gamma \cdot \text{PAR}\right)\cdot \left(1+\frac{I}{{\text{K}}_{\text{i}}}\right)}-\text{Rd}$	4.48	1.00	α = 0.044, β = 0.0001, γ = 0.0042	0.9629	131.2

EF, exponential function; HTF, hyperbolic tangent function; NHM, nonrectangular hyperbola model; RHM, rectangular hyperbolic model; BRF, binomial regression function; MM, modified model based on the rectangular hyperbolic model; NIMM, non-competitive inhibited Michaelis-Menten model; K_i_ denotes the inhibition constant; P_m_, maximum net photosynthetic rate;*e* is natural logarithm, 2.71828; a and b is constant; θ is convexity of the PI curve; α denotes the photochemical efficiency of photosynthesis at low light, *i*.*e*., the initial slope of the PI curve; β and γ are the coefficients that are independent of irradiance; Rd denotes the dark respiration rate; AIC, Akaike's information criterion.

Pollutants (metals) are harmful to plants because they inhibit various metabolic processes [41–43]. Some metal pollutants directly affect enzymes of the chlorophyll biosynthesis pathway [44–46], and some affect the proper assembly of the photosynthetic pigment-protein complexes [47, 48]. Some metalsreplace the central Mg ion in chlorophyll molecules, destroying the chlorophyll [49]. Conversely, some studies have not found that metal pollutants directly affect the biosynthesis of pigments or influence the photosynthetic machinery, and have claimed that the metal pollutants interfere with cell division and chloroplast replication, thus decreasing the number of chloroplasts and ultimately lowering the photosynthetic efficiency [50]. Thus, regardless of whether elevated concentrations of pollutants in contaminated environments bind equally well to enzymes, they will already have negatively affected plant growth and development through the inhibition of photosynthetically related enzyme activity. Our mathematical fitting results indicate that the elevated concentrations of pollutants not only inhibited α (i.e., photosynthetic potential, light use efficiency, or the slope of the PI curve), but also lowered Pn (Tables 2 and 3, Figs 1 and 3). The former (i.e., decreased α associated with increasing pollutant concentrations) suggested that the pollution decreased the activity of the photosynthetically related enzyme. Ourpot-culture experimental results showed that in *W*. *trilobata*, the pollutant (Cu^2+^) did not significantly affect the pigment content, above-ground biomass, or belowground biomass, but did significantly affect the Pn (Please see S7 Table). Our pot-culture experimental results also showed that the pollutant (phenol) significantly affected the biomass and Pn of *T*. *pratense*, but did not affect its pigment contents (Please see S8 Table). The results indicate that the pollutants acted as a non-competitive inhibitor because they varied the Pn of plants (which is equivalent to the maximum enzymatic reaction rate in the Michaelis-Menten model). Combining with the above-mentioned relationship between individual gross photosynthesis and PAR following the Michaelis-Menten model [21], that is, our results were similar to the literature [21]. And, the NIMM was suitable for reasonably predicting the relationships of Pn, PAR, and *I*.

To compare the three Michaelis kinetics (i.e., non-competitive, competitive, and un-competitive inhibition), we integrated the pollution factor into the MM in different ways, and performed mathematical fitting using our pot-culture experimental data for *T*. *pratense*. The result for un-competitive inhibited Michaelis-Menten (UIMM) kinetics was $\text{Pn}=\frac{0.081\cdot \left(1-0.0002\cdot \text{PAR}\right)\cdot \text{PAR}}{1+0.0021\cdot \text{PAR}\cdot \left(1+\frac{I}{0.80}\right)}-1.56$, R^2^ = 0.9777, and an AIC of 283.2. The result for competitive inhibited Michaelis-Menten (CIMM) kinetics was $\text{Pn}=\frac{0.073\cdot \left(1-0.0003\cdot \text{PAR}\right)\cdot \text{PAR}}{1\cdot \left(1+\frac{I}{0.470}\right)+0.0015\cdot \text{PAR}}+0.006$, R^2^ = 0.9723, and an AIC of 306.2. Both AIC values were greater than 242.5 (i.e., the AIC of the NIMM). We also tested the two models (UIMM and CIMM) using our pot-culture experimental data for *T*. *pratense*; the results are shown in Table 5. Based on the UIMM, it’s unreasonable that the φ_0_ increased but the calculated P_m_ decreased with the increasing phenol pollution. Based on the CIMM, we performed paired samples *t* test analysis, and the results showed that the calculated P_m_ was significant higher than the measured P_m_ (*t* = -5.184, *df* = 3, *P*_2-tailed_ = 0.014), i.e., the calculated P_m_ deviated greatly from the measured P_m_. So, the UIMM and CIMM were both unsuitable for predicting the relationship of Pn, PAR, and *I*. The NIMM, however, was suitable for predicting the relationship of Pn, PAR, and *I* because the calculated P_m_ values were close to the measured P_m_ (Table 3), and the fitted results were close to measured data (Fig 3).

**Table 5:** Model testing results of the un-competitive inhibited and the competitive inhibited model.

Model type	Concentrationof phenol (mg kg^-1^)	Calculated equation	Measured P_m_(μmol CO_2_ m^-2^·s^-1^)	Calculated P_m_(μmol CO_2_ m^-2^·s^-1^)	PAR_com_(μmol m^-2^ photon s^-1^)	PAR_sat_(μmol photon m^-2^ s^-1^)	φ_c_	φ_0_	R^2^
UIMM									
	0	$\text{Pn}=\frac{0.081\cdot \left(1-0.0002\cdot \text{PAR}\right)\cdot \text{PAR}}{1+0.0021\cdot \text{PAR}}-1.56$	19.5	19.4	19.3	1171.0	0.078	0.085	0.9877***
	100	$\text{Pn}=\frac{0.081\cdot \left(1-0.0002\cdot \text{PAR}\right)\cdot \text{PAR}}{1+0.0030\cdot \text{PAR}}-1.56$	15.0	14.6	19.3	1038.0	0.076	0.086	0.9851***
	200	$\text{Pn}=\frac{0.081\cdot \left(1-0.0002\cdot \text{PAR}\right)\cdot \text{PAR}}{1+0.0038\cdot \text{PAR}}-1.56$	11.3	12.0	19.3	956.0	0.075	0.087	0.9520***
	300	$\text{Pn}=\frac{0.081\cdot \left(1-0.0002\cdot \text{PAR}\right)\cdot \text{PAR}}{1+0.0047\cdot \text{PAR}}-1.56$	10.9	9.9	19.3	887.0	0.074	0.089	0.9079***
CIMM									
	0	$\text{Pn}=\frac{0.073\cdot \left(1-0.0003\cdot \text{PAR}\right)\cdot \text{PAR}}{1+0.0015\cdot \text{PAR}}+0.006$	19.5	20.4	0.008	966.3	0.073	0.073	0.9650***
	100	$\text{Pn}=\frac{0.073\cdot \left(1-0.0003\cdot \text{PAR}\right)\cdot \text{PAR}}{1.70+0.0015\cdot \text{PAR}}+0.006$	15.0	16.8	0.014	1143.6	0.043	0.043	0.8973***
	200	$\text{Pn}=\frac{0.073\cdot \left(1-0.0003\cdot \text{PAR}\right)\cdot \text{PAR}}{2.40+0.0015\cdot \text{PAR}}+0.006$	11.3	13.6	0.020	1220.1	0.030	0.030	0.8155***
	300	$\text{Pn}=\frac{0.073\cdot \left(1-0.0003\cdot \text{PAR}\right)\cdot \text{PAR}}{3.13+0.0015\cdot \text{PAR}}+0.006$	10.9	12.2	0.026	1318.8	0.023	0.023	0.7567***

UIMM is the un-competitive inhibited Michaelis-Menten; CIMM is the competitive inhibited Michaelis-Menten; PAR_sat_ is the light saturation point; PAR_com_ is the light compensation point; P_m_ is the maximum photosynthetic rate; φ_c_ is the quantum efficiency at PAR_com_; φ_0_ is the intrinsic quantum efficiency; ${\text{PAR}}_{\text{com}}=\frac{\text{Rd}}{\alpha }$, φ_0_ = α∙[1+(β+γ)∙PAR_com_],$\varphi_{\text{c}}=\alpha \cdot \frac{1+\left(\beta +\gamma \right)\cdot {\text{PAR}}_{\text{com}}-\beta \cdot \gamma \cdot {\text{PAR}}_{\text{com}}^{2}}{{\left(1+\gamma \cdot {\text{PAR}}_{\text{com}}\right)}^{2}}$, ${\text{PAR}}_{\text{sat}}=\frac{\sqrt{\frac{\left(\beta +\gamma \right)\cdot \left(1+\gamma \cdot {\text{PAR}}_{\text{com}}\right)}{\beta }}-1}{\gamma }$, ${\text{P}}_{\text{m}}=\frac{\alpha \cdot \left(1-\beta \cdot {\text{PAR}}_{\text{sat}}\right)\cdot {\text{PAR}}_{\text{sat}}}{1+\gamma \cdot {\text{PAR}}_{\text{sat}}}-\text{Rd}$;

*** means significant at *P* ≤ 0.001.

Interestingly, pollutants play a role in the inhibition of photosynthetically related enzyme activity; the K_i_ decreased with the combination of the pollutant with the photosynthetically related enzyme. The mathematical fitting results (Table 2) indicate that *W*. *trilobata* is tolerant of Cu pollution [51].

Finally, we put forward a perspective that the field investigation still needs to be further done for model validation. The published results [22–25] and the present study showed that the pollution factor could affect the PI curve in controlled experiment. In natural environment, many other uncontrolled variables such as temperature, humidity, CO_2_ concentrations and so on, can also affect photosynthetic parameters. Therefore, it is important to justify and reveal the accuracy of the NIMM in practice.

## Supporting Information

S1 Table Effect of Pb^2+^ on the Pn of *Zea mays*. (DOCX)

S2 Table Effect of Cu^2+^ on the Pnof *Citrus sinensis* Osbeck. (DOCX)

S3 Table Effect of Cd^2+^ on the Pnof *Zea mays*. (DOCX)

S4 Table Effect of Al^3+^ on the Pnof *Plantago asiatica*. (DOCX)

S5 Table Effect of phenol on the Pn of *Trifolium pratense* L. (DOCX)

S6 Table Effect of CuSO_4_·5H_2_O on the Pn of *Wedelia trilobata*. (DOCX)

S7 Table Effect of Cu^2+^ on *W*. *trilobata*. (DOCX)

S8 Table Effect of phenol on *T*. *pratense* L. (DOCX)
