# Supplementary material for: Plant Photosynthesis-Irradiance Curve Responses to Pollution Show Non-Competitive Inhibited Michaelis Kinetics
Source: PLoS One. 2015 Nov 12;10(11):e0142712. doi: 10.1371/journal.pone.0142712 (PMC4642952; doi:10.1371/journal.pone.0142712)
Supplement: S1 Table — (DOCX) [file pone.0142712.s001.docx]

| **S1 Table. Effect of Pb^2+^ on the Pn of *Zea mays*** | | | |
| --- | --- | --- | --- |
| PAR | 0 mmol·L^-1^ | 0.25 mmol·L^-1^ | 0.5 mmol·L^-1^ |
| 0 | -3.3 | -2.2 | -1.6 |
| 50 | 0.0 | 0.2 | -1.3 |
| 100 | 1.8 | 1.1 | -0.2 |
| 150 | 3.3 | 1.8 | 0.5 |
| 200 | 5.1 | 3.2 | 1.0 |
| 300 | 8.8 | 4.8 | 1.9 |
| 400 | 11.9 | 6.5 | 2.7 |
| 600 | 17.3 | 8.8 | 2.9 |
| 800 | 21.2 | 9.7 | 3.7 |
| 1000 | 23.7 | 10.3 | 4.0 |
| 1200 | 25.5 | 10.5 | 4.1 |
| 1400 | 26.3 | 10.7 | 4.1 |
| 1600 | 26.3 | 10.3 | 4.1 |

Note: where PAR is photosynthetically active radiation (μmol photon m^-2^ s^-1^), Pn is net photosynthetic rate (μmol CO_2_ m^-2^ s^-1^).
