# Supplementary material for: Plant Photosynthesis-Irradiance Curve Responses to Pollution Show Non-Competitive Inhibited Michaelis Kinetics
Source: PLoS One. 2015 Nov 12;10(11):e0142712. doi: 10.1371/journal.pone.0142712 (PMC4642952; doi:10.1371/journal.pone.0142712)
Supplement: S2 Table — (DOCX) [file pone.0142712.s002.docx]

| **S2 Table. Effect of Cu^2+^ on the Pn of *Citrus sinensis* Osbeck** | | | | | |
| --- | --- | --- | --- | --- | --- |
| PAR | 0 μmol L^-1^ | 0.1 μmol L^-1^ | 5 μmol L^-1^ | 20 μmol L^-1^ | 40 μmol L^-1^ |
| 0 | -0.4 | -0.5 | -0.8 | -1.0 | -1.0 |
| 50 | 0.4 | 0.5 | 0.3 | 0.2 | 0.0 |
| 100 | 0.9 | 1.0 | 0.4 | 0.3 | 0.2 |
| 290 | 2.8 | 3.1 | 2.0 | 1.0 | 0.5 |
| 510 | 4.0 | 4.6 | 3.2 | 1.7 | 1.0 |
| 700 | 5.1 | 5.7 | 4.0 | 2.1 | 1.2 |
| 1000 | 6.6 | 7.2 | 5.0 | 2.6 | 1.1 |
| 1200 | 7.2 | 7.9 | 5.4 | 2.6 | 1.1 |
| 1500 | 7.1 | 7.9 | 5.4 | 2.5 | 1.0 |
| 2000 | 6.1 | 6.8 | 5.0 | 2.2 | 0.8 |

Note: where PAR is photosynthetically active radiation (μmol photon m^-2^ s^-1^), Pn is net photosynthetic rate (μmol CO_2_ m^-2^ s^-1^).
