# Supplementary material for: Plant Photosynthesis-Irradiance Curve Responses to Pollution Show Non-Competitive Inhibited Michaelis Kinetics
Source: PLoS One. 2015 Nov 12;10(11):e0142712. doi: 10.1371/journal.pone.0142712 (PMC4642952; doi:10.1371/journal.pone.0142712)
Supplement: S3 Table — (DOCX) [file pone.0142712.s003.docx]

| **S3 Table. Effect of Cd^2+^ on the Pn of *Zea mays*** | | | | | | |
| --- | --- | --- | --- | --- | --- | --- |
| PAR | 0.32 mg Kg^-1)^ | 1 mg Kg^-1^ | 5 mg Kg^-1^ | 15 mg Kg^-1^ | 50 mg Kg^-1^ | 100 mg Kg^-1^ |
| 5 | -2.1 | -1.2 | -2.2 | -1.9 | -1.7 | -2.9 |
| 50 | 1.4 | 2.2 | 1.6 | 0.5 | 1.0 | 0.2 |
| 100 | 3.3 | 4.1 | 3.1 | 2.1 | 2.8 | 1.2 |
| 150 | 4.8 | 6.2 | 5.2 | 3.1 | 5.0 | 2.4 |
| 200 | 6.7 | 7.6 | 6.7 | 5.0 | 5.9 | 3.3 |
| 300 | 8.8 | 10.3 | 8.8 | 7.8 | 7.4 | 4.8 |
| 400 | 10.7 | 16.2 | 15.0 | 9.8 | 9.7 | 6.0 |
| 800 | 14.0 | 23.3 | 19.7 | 13.3 | 12.1 | 7.8 |
| 1000 | 15.0 | 25.5 | 20.9 | 14.1 | 13.6 | 9.3 |
| 1200 | 15.5 | 27.4 | 21.9 | 14.5 | 14.3 | 8.8 |
| 1800 | 16.9 | 29.1 | 23.6 | 15.3 | 15.0 | 9.0 |
| 2000 | 16.9 | 29.1 | 23.4 | 16.2 | 14.7 | 9.3 |

Note: where PAR is photosynthetically active radiation (μmol photon m^-2^ s^-1^), Pn is net photosynthetic rate (μmol CO_2_ m^-2^ s^-1^).
