# Supplementary material for: Plant Photosynthesis-Irradiance Curve Responses to Pollution Show Non-Competitive Inhibited Michaelis Kinetics
Source: PLoS One. 2015 Nov 12;10(11):e0142712. doi: 10.1371/journal.pone.0142712 (PMC4642952; doi:10.1371/journal.pone.0142712)
Supplement: S4 Table — (DOCX) [file pone.0142712.s004.docx]

| **S4 Table. Effect of Al^3+^ on the Pn of *Plantago Asiatica*** | | | | | |
| --- | --- | --- | --- | --- | --- |
| PAR | CK (0 mg L^-1^) | 100 mg L^-1^ | 500 mg L^-1^ | 800 mg L^-1^ | 2000 mg L^-1^ |
| 0 | -1.5 | -0.4 | -1.5 | -1.5 | -1.2 |
| 50 | 0.7 | 1.6 | 1.0 | 0.7 | 0.1 |
| 100 | 2.7 | 3.8 | 2.7 | 2.3 | 1.1 |
| 150 | 4.8 | 5.8 | 4.1 | 3.9 | 1.9 |
| 200 | 6.8 | 7.4 | 5.6 | 5.6 | 2.3 |
| 300 | 9.7 | 10.8 | 7.7 | 7.6 | 2.7 |
| 400 | 13.3 | 14.2 | 10.0 | 9.8 | 2.9 |
| 500 | 16.7 | 17.9 | 12.2 | 12.0 | 3.0 |
| 600 | 18.8 | 20.8 | 14.2 | 13.3 | 3.3 |
| 800 | 22.3 | 24.7 | 16.2 | 14.9 | 3.3 |
| 1000 | 23.7 | 26.1 | 16.9 | 15.4 | 4.1 |
| 1200 | 24.7 | 27.5 | 16.9 | 15.0 | 4.4 |
| 1400 | 25.2 | 28.1 | 17.2 | 15.0 | 4.5 |
| 1600 | 25.2 | 28.3 | 17.3 | 15.1 | 4.6 |

Note: where PAR is photosynthetically active radiation (μmol photon m^-2^ s^-1^), Pn is net photosynthetic rate (μmol CO_2_ m^-2^ s^-1^).
