# Supplementary material for: Plant Photosynthesis-Irradiance Curve Responses to Pollution Show Non-Competitive Inhibited Michaelis Kinetics
Source: PLoS One. 2015 Nov 12;10(11):e0142712. doi: 10.1371/journal.pone.0142712 (PMC4642952; doi:10.1371/journal.pone.0142712)
Supplement: S5 Table — (DOCX) [file pone.0142712.s005.docx]

| **S5 Table. Effect of phenol on the Pn of *Trifolium pratense* L.** | | | | |
| --- | --- | --- | --- | --- |
| PAR | CK (0 mg Kg^-1^) | 100 mg Kg^-1^ | 200 mg Kg^-1^ | 300 mg Kg^-1^ |
| 1600 | 18.5 | 14.9 | 10.3 | 10.4 |
| 1500 | 18.8 | 15.0 | 10.4 | 10.7 |
| 1400 | 19.1 | 15.0 | 10.7 | 10.8 |
| 1300 | 19.3 | 14.8 | 10.7 | 10.8 |
| 1200 | 19.4 | 14.6 | 10.9 | 10.9 |
| 1100 | 19.4 | 14.9 | 11.1 | 10.7 |
| 1000 | 19.5 | 14.7 | 11.0 | 10.9 |
| 900 | 19.5 | 14.7 | 11.2 | 10.6 |
| 800 | 19.2 | 14.7 | 11.3 | 10.3 |
| 700 | 18.7 | 14.6 | 10.9 | 10.2 |
| 600 | 18.5 | 14.3 | 10.8 | 9.8 |
| 500 | 17.6 | 13.7 | 10.3 | 9.2 |
| 400 | 16.1 | 12.8 | 9.5 | 8.2 |
| 300 | 13.8 | 10.6 | 8.3 | 6.8 |
| 200 | 9.8 | 7.5 | 6.2 | 4.9 |
| 100 | 5.1 | 3.7 | 3.4 | 2.4 |
| 50 | 2.2 | 1.7 | 1.5 | 0.9 |
| 0 | -0.6 | -1.3 | -0.6 | -0.7 |

Note: where PAR is photosynthetically active radiation (μmol photon m^-2^ s^-1^), Pn is net photosynthetic rate (μmol CO_2_ m^-2^ s^-1^).
