# Supplementary material for: Plant Photosynthesis-Irradiance Curve Responses to Pollution Show Non-Competitive Inhibited Michaelis Kinetics
Source: PLoS One. 2015 Nov 12;10(11):e0142712. doi: 10.1371/journal.pone.0142712 (PMC4642952; doi:10.1371/journal.pone.0142712)
Supplement: S6 Table — (DOCX) [file pone.0142712.s006.docx]

| **S6 Table. Effect of CuSO_4_·5H_2_O on the Pn of** ***Wedelia trilobata*** | | | | |
| --- | --- | --- | --- | --- |
| PAR | CK (0 mg Kg^-1^) | 500mg Kg^-1^ | 1000mg Kg^-1^ | 2000mg Kg^-1^ |
| 50 | 0.9 | 0.7 | 0.6 | 0.5 |
| 100 | 1.8 | 1.9 | 1.7 | 1.5 |
| 200 | 3.6 | 3.2 | 2.9 | 2.9 |
| 300 | 4.3 | 4.2 | 4.3 | 4.1 |
| 400 | 4.9 | 4.6 | 4.5 | 4.6 |
| 500 | 5.6 | 5.6 | 5.3 | 4.6 |
| 600 | 5.9 | 5.6 | 5.4 | 4.7 |
| 700 | 6.5 | 5.5 | 5.2 | 5.1 |
| 800 | 6.7 | 5.3 | 5.1 | 4.9 |
| 900 | 6.3 | 5.3 | 5.2 | 5 |
| 1000 | 6.5 | 5.3 | 5.2 | 5 |
| 1100 | 6.5 | 5.6 | 5.3 | 4.9 |
| 1200 | 6.7 | 5.6 | 5.2 | 5 |
| 1300 | 6.7 | 5.6 | 5.4 | 5.1 |
| 1400 | 6.7 | 5.4 | 5.2 | 5.2 |
| 1500 | 6.4 | 5.6 | 5.6 | 4.9 |
| 1600 | 6.3 | 5.4 | 5.2 | 5 |

Note: where PAR is photosynthetically active radiation (μmol photon m^-2^ s^-1^), Pn is net photosynthetic rate (μmol CO_2_ m^-2^ s^-1^).
