# Supplementary material for: Plant Photosynthesis-Irradiance Curve Responses to Pollution Show Non-Competitive Inhibited Michaelis Kinetics
Source: PLoS One. 2015 Nov 12;10(11):e0142712. doi: 10.1371/journal.pone.0142712 (PMC4642952; doi:10.1371/journal.pone.0142712)
Supplement: S7 Table — (DOCX) [file pone.0142712.s007.docx]

| **S7 Table. Effect of Cu^2+^ on *W. trilobata*** | | | | |
| --- | --- | --- | --- | --- |
| CuSO_4_·5H_2_O in soil (mg·kg^-1^) | 0 | 500 | 1000 | 2000 |
| Net photosynthetic rate (μmol m^-2^ s^-1^), n=15 | 5.2 ± 0.2^a^ | 3.6 ± 0.4^b^ | 2.3 ± 0.3^c^ | 2.68 ± 0.2^c^ |
| Transpiration rate (m mol m^-2^ s^-1^) , n=15 | 0.41 ± 0.02^a^ | 0.35 ± 0.03^a^ | 0.27 ± 0.01^b^ | 0.26 ± 0.01^b^ |
| Stomatal conductance (m mol m^-2^ s^-1^) , n=15 | 14.5 ± 0.8^a^ | 12.0 ± 1.1^b^ | 8.6 ± 0.4^c^ | 8.2 ± 0.4^c^ |
| Chlorophyll a (μg·g^-1^), n=5 | 212.3 ± 31.5a | 223.1 ± 46.8a | 242.4 ± 46.1a | 182.7 ± 45.1a |
| Chlorophyll b (μg·g^-1^), n=5 | 92.8 ± 16.7a | 82.4 ± 13.9a | 113.3 ± 27.2a | 90.8 ± 19.3a |
| Ground biomass （g per plant, dry weight）, n=6 | 1.32 ± 0.21a | 1.58 ± 0.34 a | 1.25 ± 0.39 a | 1.65 ± 0.12a |
| Under-ground biomass (g per plant, dry weight), n=6 | 1.51 ± 0.25a | 1.72 ± 0.25a | 1.63 ± 0.31a | 1.71 ± 0.42a |

Note: 1) Data shows mean ± SE, the same letter means no significant different at *P* ≤ 0.05 within the same row, the different letter means difference between groups at *P* ≤ 0.05 within the same row, compared by Duncan’s multiple range test;

2) Net photosynthetic rate, Transpiration rate, and Stomatal conductance were measured using Ciras-2 portable photosynthesis system (PP systems, UK) with a LED radiation source set to 800 μmol m^-2^ s^-1^;

3) The chlorophylls were measured according to Arnon method. Briefly, fresh leaf was cut into pieces of ≈0.2 g, and then it was dipped into 10 mL of 80% acetone solution and shacked in dark at room temperature for 48 h. The absorption spectrum was set at 663 and 645 nm with UV-1601 respectively (Shimadzu, Japan). Chlorophyll a and b were respectively calculated as follows:

Chlorophyll a (μg·g^-1^) = C_a_/w;

Chlorophyll b (μg·g^-1^) = C_b_/w;

where w is fresh leaf piece weight (g), C_a_ = 12.7 × OD_663_ - 2.69 × OD_645_, C_b_ = 22.9 × OD_645_ - 4.68 × OD_663_, where OD_663_ and OD_645_ are absorbances at 663 and 645 nm, respectively.

4) The biomass was measured by weighting method.
