# Supplementary material for: Plant Photosynthesis-Irradiance Curve Responses to Pollution Show Non-Competitive Inhibited Michaelis Kinetics
Source: PLoS One. 2015 Nov 12;10(11):e0142712. doi: 10.1371/journal.pone.0142712 (PMC4642952; doi:10.1371/journal.pone.0142712)
Supplement: S8 Table — (DOCX) [file pone.0142712.s008.docx]

| **S8 Table. Effect of phenol on *T. pratense* L.** | | | | |
| --- | --- | --- | --- | --- |
| Phenol in soil (mg·kg^-1^) | 0 | 100 | 200 | 300 |
| Net photosynthetic rate (μmol m^-2^ s^-1^), n=15 | 13.9 ± 0.7^a^ | 12.2 ± 0.3^b^ | 10.8 ± 0.7b^b^ | 6.48 ± 0.6^c^ |
| Transpiration rate (m mol m^-2^ s^-1^) , n=15 | 0.45 ± 0.02^a^ | 0.34 ± 0.03^a^ | 0.32 ± 0.02^b^ | 0.27 ± 0.01^b^ |
| Stomatal conductance (m mol m^-2^ s^-1^) , n=15 | 13.5 ± 0.8^a^ | 12.3 ± 1.1^a^ | 9.3 ± 0.4^b^ | 9.2 ± 0.6^b^ |
| Chlorophyll a (μg·g^-1^), n=5 | 229.3 ± 43.6^a^ | 215.4 ± 58.1^a^ | 244.3 ± 48.5^a^ | 234.6 ± 44.6^a^ |
| Chlorophyll b (μg·g^-1^), n=5 | 104.1 ± 23.9^a^ | 96.9 ± 19.4^a^ | 117.4 ± 22.3^a^ | 113.1 ± 18.1^a^ |
| Ground biomass （g per plant, dry weight）, n=6 | 3.21 ± 0.31^a^ | 2.11 ± 0.12^b^ | 1.32 ± 0.31^c^ | 0.91 ± 0.31^cd^ |
| Under-ground biomass (g per plant, dry weight), n=6 | 3.03 ± 0.27^a^ | 2.24 ± 0.21^b^ | 1.83 ± 0.16^bc^ | 1.51 ± 0.25^c^ |

Note: 1) Data shows mean ± SE, the same letter means no significant different at *P* ≤ 0.05 within the same row, the different letter means difference between groups at *P* ≤ 0.05 within the same row, compared by Duncan’s multiple range test;

2) Net photosynthetic rate, Transpiration rate, and Stomatal conductance were measured using Ciras-2 portable photosynthesis system (PP systems, UK) with a LED radiation source set to 800 μmol m^-2^ s^-1^;

3) The chlorophylls were measured according to Arnon method. Briefly, fresh leaf was cut into pieces of ≈0.2 g, and then it was dipped into 10 mL of 80% acetone solution and shacked in dark at room temperature for 48 h. The absorption spectrum was set at 663 and 645 nm with UV-1601 respectively (Shimadzu, Japan). Chlorophyll a and b were respectively calculated as follows:

Chlorophyll a (μg·g^-1^) = C_a_/w;

Chlorophyll b (μg·g^-1^) = C_b_/w;

where w is fresh leaf piece weight (g), C_a_ = 12.7 × OD_663_ - 2.69 × OD_645_, C_b_ = 22.9 × OD_645_ - 4.68 × OD_663_, where OD_663_ and OD_645_ are absorbances at 663 and 645 nm, respectively.

4) The biomass was measured by weighting method.
